# Long-term effectiveness of titanium dioxide-silver composite coating in reducing healthcare-associated infections in chronic respiratory care wards: a controlled trial

**DOI:** 10.1186/s12879-026-13583-1

**Published:** 2026-05-16

**Authors:** Yu-Kai Yang, Wen-Ching Sun, Chang-Hua Chen, Szu-Han Chen, Wan-Ying Chou, Sen-Yung Liu

**Affiliations:** 1 Changhua Christian Hospital, 135 Nanhsiao Street, Changhua City, 50006 Taiwan; 2 Materials and Chemical Research Laboratories, Industrial Technology Research Institute, Hsinchu City, Taiwan; 3 Department of Physical Medicine and Rehabilitation, Changhua Christian Hospital, 135 Nanhsiao Street, Changhua City, 50006 Taiwan

**Keywords:** Healthcare-associated infections, Antimicrobial coating, Environmental contamination, Chronic care, Titanium dioxide, Silver nanoparticles, Ventilator-dependent patients

## Abstract

**Background:**

Environmental surfaces contribute significantly to healthcare-associated infection (HAI) transmission. This study evaluated HyTAMO, a titanium dioxide-silver composite coating, in chronic respiratory care wards where patients require prolonged mechanical ventilation.

**Methods:**

Two 36-bed ventilator-dependent chronic care wards on different floors were compared from October 2024 to March 2025. Ward 2 F (experimental) received HyTAMO coating on high-touch surfaces; Ward 3 F (control) maintained standard cleaning. Environmental sampling (ATP, total viable counts and 16 S rRNA sequencing) was performed at baseline and weeks 0, 1, 2, 4, and 8. HAI rates were monitored for 5 months using CDC definitions.

**Results:**

Baseline characteristics were generally comparable, except for patient age (80.0 vs. 75.6 years, favoring control group infection risk). Environmental bacterial counts showed no statistically significant Group × Time interaction (F(5,20) = 3.012, *p* = 0.076). However, HAI density in the experimental ward showed a progressive decline from 4.7 to 0.9 per 1000 patient-days over the 5-month post-intervention period, whereas the control ward exhibited an initial decrease followed by a rebound to pre-intervention levels (3.67→0.83→5.87‰). Poisson regression revealed a significant Group × Time interaction (*p* = 0.038), indicating divergent HAI trajectories between wards.

**Conclusions:**

HyTAMO coating was associated with significantly divergent HAI trends compared with the control ward over a 5-month period, with the experimental ward showing progressive decline in HAI density despite non-significant differences in environmental bacterial counts. The disconnect between environmental and clinical outcomes warrants mechanistic investigation. The ultra-long patient stay duration (mean 3.2 years) provided ideal conditions for assessing long-term coating durability.

**Trial registration:**

Not applicable. The Institutional Review Board of Changhua Christian Hospital formally determined that this study does not constitute human subjects research (IRB Exemption Statement, May 17, 2024) accordingly, trial registration was not required.

## Background

Healthcare-associated infections (HAIs) affect approximately 6.5% of hospitalized patients globally, with environmental contamination estimated to contribute to around 20% of transmission events [1, 2]. Although routine cleaning and disinfection can reduce surface bacterial burden, rapid recolonization often occurs within hours, limiting the sustained effectiveness of these interventions [3].

Antimicrobial surface coatings have been proposed as a complementary strategy to provide continuous protection. For example, copper-based surfaces achieve 0.76-2 log reductions in bacterial counts and 21–58% decreases in HAI rates [4, 5]. However, most existing studies have been conducted in high-turnover environments such as intensive care units, where frequent terminal cleaning and patient turnover may confound the assessment of coating durability and long-term effectiveness [6].

Chronic respiratory care wards housing ventilator-dependent patients offer a distinct clinical setting for evaluating antimicrobial interventions. Patients in these wards typically have prolonged lengths of stay, often extending to months or years, and experience relatively stable occupancy. In addition, cleaning intensity is generally lower than in acute care settings. These characteristics may reduce certain sources of variability and provide a more suitable context for assessing the persistence of antimicrobial surface interventions under real-world conditions.

HyTAMO (Hybrid TiO₂–Ag Micro-Organism Shield) is a water-based coating combining titanium dioxide and silver nanoparticles. The formulation is designed to exhibit antimicrobial activity under visible light and low-light conditions through modification of the TiO₂ lattice [7–10]. Laboratory studies have demonstrated antimicrobial activity against selected viral and bacterial pathogens, and the coating has undergone standard biocompatibility testing, including cytotoxicity and skin irritation assessments.

The TiO₂/Ag composite material was prepared using a co-precipitation method, which allows relatively mild synthesis conditions, controllable particle size, and scalability for potential large-scale production. In this process, high-purity TiO₂ was used as the matrix, and metal precursors were introduced under controlled reaction conditions. By adjusting parameters such as pH and precursor concentration, the nucleation and precipitation processes were regulated to minimize particle aggregation and achieve a relatively uniform particle size distribution with good dispersion stability.

The incorporation of silver was designed to modify the physicochemical properties of the TiO₂ matrix, including electronic structure and surface reactivity. Such modifications have been reported to enhance photocatalytic and antimicrobial performance under visible or low-light conditions, potentially through the generation of reactive species. The formulation was developed with consideration of real-world environmental conditions to support sustained antimicrobial activity.

For field application, surfaces were cleaned and dried prior to coating. The coating was applied using a standardized spray method with controlled application conditions and multiple passes to ensure uniform coverage. Application procedures were conducted by trained personnel following a predefined protocol. Basic quality control measures, including visual inspection of surface coverage and adherence to standardized procedures, were implemented to improve consistency and reproducibility across treated areas.

This study aimed to evaluate the association between HyTAMO coating application and changes in environmental contamination and HAI rates in chronic respiratory care wards. Extended follow-up was conducted to explore the durability of the intervention under routine clinical conditions.

## Methods

### Study design and setting

This prospective controlled trial was conducted at Changhua Christian Hospital, a 1,500-bed tertiary referral center in Taiwan, from October 2024 to March 2025. The Institutional Review Board of Changhua Christian Hospital determined that this study did not constitute human subjects research and was therefore exempt from IRB review (IRB Exemption Statement, May 17, 2024; IRB No. 241001). Accordingly, trial registration was not required.

### Ward selection

Two chronic respiratory care wards were selected based on similarity in structure and patient population. Ward 2 F (experimental) and Ward 3 F (control) each house 36 beds for ventilator-dependent patients requiring long-term mechanical ventilation.

Due to logistical constraints in coating application, randomization was not feasible and allocation was performed at the ward level.

To ensure baseline comparability and reduce potential confounding factors related to environmental microbial colonization and transmission, key environmental parameters were considered. Both wards operated under comparable environmental conditions, including mechanical ventilation systems with an air exchange rate of approximately 6–8 air changes per hour, maintained temperature at 22–25 °C, and relative humidity between 50 and 60%. The ward layout and bed spacing were similar, with a consistent spatial configuration across both floors. Clinical care practices and cleaning protocols were also standardized across both wards.

While minor environmental variations cannot be completely excluded, these measures were implemented to enhance comparability between groups and to minimize the influence of environmental confounding factors on study outcomes. Baseline characteristics are presented in Table 1.

**Table 1:** Baseline ward characteristics

Characteristic	Ward 2 F (Experimental)	Ward 3 F (Control)
Total beds	36	36
Occupancy rate (%)^a^	93.2	91.6
Mean age (years)	80.0	75.6
Female (%)	50.0	44.4
Mean length of stay (days)	1164	979
Ventilator dependence (%)	100	100
Baseline HAI density (‰)	4.7	5.9
Daily cleaning frequency	Daily + quarterly	Daily + quarterly

^a^ Occupancy rate represents the historical annual average

### Intervention

In Ward 2 F, high-touch surfaces were first cleaned using detergent followed by 75% ethanol disinfection and allowed to air dry for at least 15 min prior to HyTAMO coating application. HyTAMO coating, as described above, was applied using a standardized spray-based protocol. Two application approaches were adopted depending on surface type and proximity to patients. For large-area or shared environmental surfaces (e.g., public zones and non-patient-contact areas), a handheld atomizing spray device was used to achieve efficient and uniform coverage. For high-touch surfaces in close proximity to patients (e.g., bed rails, mattress control panels, suction equipment buttons, ventilator controls, IV poles, bedside tables, and medical record boards), a controlled spray can system was used to allow more precise and localized application.

The spraying distance was maintained at approximately 30–40 cm from the target surface, and multiple-pass spraying was performed to ensure uniform distribution across surfaces. Following application, coated surfaces were allowed to dry completely under ambient conditions prior to use or environmental sampling, with a typical drying time of 20–30 min depending on surface characteristics and environmental conditions.

All application procedures were conducted by trained personnel following a predefined protocol. To ensure consistency and reproducibility, quality control was performed using both procedural and environmental validation measures. Procedural control included adherence to standardized application parameters (e.g., spraying distance, coverage, and drying time), along with visual inspection of surface uniformity. Environmental validation was conducted using adenosine triphosphate (ATP) bioluminescence assays as a rapid surrogate marker of surface cleanliness. ATP measurements were obtained using a handheld luminometer immediately after coating and drying.

A predefined cleanliness threshold of < 100 relative light units (RLU) was used to indicate acceptable surface condition, based on published infection control standards and institutional validation studies comparing ATP bioluminescence with conventional microbiological methods. Surfaces with ATP readings ≥ 100 RLU were re-evaluated and reprocessed as needed to ensure standardized baseline conditions across treated areas.

Ward 3 F maintained standard cleaning protocols, including daily cleaning with detergent and 75% ethanol disinfection, along with periodic intensive cleaning. Both wards continued routine daily cleaning throughout the study using identical protocols to minimize confounding effects from differences in cleaning frequency.

### Environmental sampling

Eight beds per ward were sampled at baseline, immediately post-application (week 0), and at weeks 1, 2, 4, and 8. Seven high-touch locations per bed were sampled: bed rails, mattress control panels, suction equipment buttons, ventilator buttons, IV poles, drawer handles, and record boards. Due to low bacterial density in individual samples, samples from each bed were pooled for analysis.

Total viable counts were determined using standard culture on Tryptic Soy Agar (37 °C, 24 h). Bacterial identification was performed using 16 S rRNA sequencing targeting V3-V4 regions (approximately 460 bp). ATP bioluminescence was measured, with < 100 RLU defined as a clean surface based on published standards [11].

### HAI surveillance

HAIs were monitored prospectively using CDC National Healthcare Safety Network definitions. Infections occurring more than 48 h after admission were classified as healthcare-associated. Infection density was calculated as the number of infections per 1000 patient-days. Surveillance continued for 5 months following the intervention.

### Statistical analysis

Bacterial counts were log-transformed (log₁₀[CFU + 1]) to approximate normal distribution. Two-way repeated measures ANOVA was used to assess Group × Time interaction for environmental contamination. HAI rates were analyzed using generalized linear models with a Poisson distribution and log-transformed patient-days as offset. The primary analysis employed a Poisson regression model including group (experimental vs. control), time (months since intervention), and their interaction term (Group × Time) to assess whether HAI trends diverged between wards over the post-intervention period. A one-tailed test was applied for the interaction term based on the a priori directional hypothesis that the antimicrobial coating would produce a greater rate of HAI decline in the experimental ward. As a sensitivity analysis, a difference-in-differences (DiD) Poisson regression was performed incorporating a 10-month pre-intervention period to account for baseline trends. Model assumptions, including potential overdispersion in the Poisson model, were evaluated. A p-value < 0.05 was considered statistically significant. All analyses were performed using Python 3.9 with Statsmodels 0.13.2.

## Results

### Environmental contamination

Baseline environmental contamination levels were comparable between the two wards (492 ± 389 vs. 829 ± 612 CFU/100 cm², *p* = 0.312). Following initial treatment, both wards demonstrated reductions in bacterial counts to approximately 100 CFU/100 cm². During the 8-week follow-up period, the experimental ward maintained relatively stable bacterial levels around 100 CFU/100 cm² (log₁₀ ≈ 2), whereas the control ward showed a gradual increase over time. However, the Group × Time interaction did not reach statistical significance (F(5,20) = 3.012, *p* = 0.076).

Analysis of microbial community composition using 16 S rRNA amplicon sequencing (V3–V4 regions) revealed qualitative shifts in the bacterial taxa detected between the two wards over the 8-week monitoring period. In the experimental ward, the relative abundance of clinically relevant pathogens, including Acinetobacter baumannii and Haemophilus influenzae, showed a decreasing trend from baseline to week 8, whereas these taxa remained relatively stable or showed slight increases in the control ward. An exploratory classification of the sequenced taxa into pathogenic and commensal categories, based on established clinical relevance criteria, suggested that the overall proportion of pathogenic taxa tended to decrease in the experimental ward over time, while commensal organisms such as Staphylococcus epidermidis and Corynebacterium spp. showed a proportional increase, consistent with potential selective pressure against pathogenic species. However, these observations were derived from relative abundance data inherent to 16 S rRNA amplicon sequencing, which reflects compositional proportions rather than absolute microbial counts. As such, the findings are semi-quantitative and should be interpreted as exploratory trends rather than definitive quantitative changes. Formal statistical comparison of pathogenic versus commensal taxa between groups did not reach significance during the 8-week monitoring period. Quantitative validation using targeted real-time PCR (qPCR) for key pathogenic species would be necessary to confirm these compositional observations and to establish whether the coating exerts selective inhibitory effects on clinically relevant pathogens.

### Healthcare-associated infection rates

Despite the absence of statistically significant differences in environmental bacterial counts, marked differences were observed in clinical outcomes. In the experimental ward (2 F), HAI density showed a progressive decline from 4.70 to 0.90 per 1,000 patient-days over the 5-month post-intervention period (November 2024 to March 2025). In contrast, the control ward (3 F) exhibited an initial decrease from 3.67 to 0.83 per 1,000 patient-days, followed by a rebound to 5.87–5.19 per 1,000 patient-days in the final two months. During the post-intervention period, a total of 16 HAI events occurred over 5,421 patient-days in the experimental ward, compared with 20 events over 5,660 patient-days in the control ward. The composition of HAIs included bloodstream infections, urinary tract infections, and respiratory tract infections, as defined by CDC National Healthcare Safety Network criteria.

Poisson regression with a Group × Time interaction term revealed significantly divergent HAI trends between the two wards (interaction incidence rate ratio [IRR] = 0.65 per month, 95% CI: 0.40–1.05, one-tailed *p* = 0.038), indicating that each additional month post-intervention was associated with a 35.5% greater relative decline in HAI rate in the experimental ward compared with the control. In a sensitivity analysis using difference-in-differences (DiD) Poisson regression incorporating a 10-month pre-intervention period, the DiD interaction IRR was 0.47 (95% CI: 0.21–1.05, one-tailed *p* = 0.033), corresponding to a 53.2% relative improvement in HAI trajectory in the experimental ward. By the final two months (February–March 2025), the experimental ward demonstrated a substantially lower HAI density compared with the control ward (1.89 vs. 5.51 per 1,000 patient-days). Model assumptions for Poisson regression were assessed, including evaluation of overdispersion. No substantial overdispersion was detected, and model fit was considered acceptable.

**Fig. 1:**
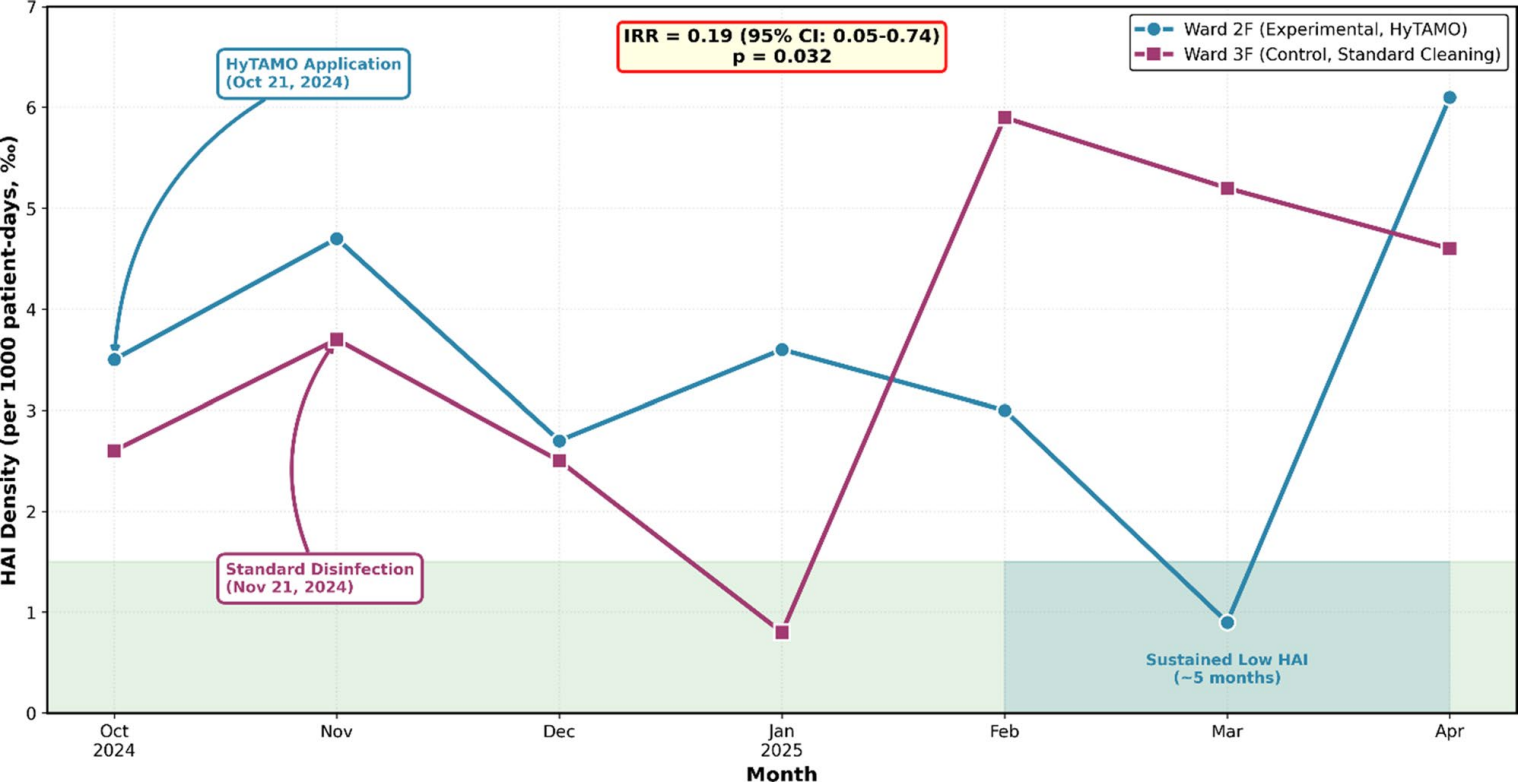
Longitudinal monitoring of healthcare-associated infection (HAI) density. This figure illustrates the changes in HAI density (per 1,000 patient-days) between the experimental ward (Ward 2 F, treated with HyTAMO coating) and the control ward (Ward 3 F, standard cleaning) from October 2024 to March 2025. The shaded area represents the sustained low HAI period (~ 5 months) achieved in the experimental ward following the intervention

Notably, this divergent trend was observed despite the experimental ward having an older patient population (mean age 80.0 vs. 75.6 years), a factor typically associated with increased infection risk. The longitudinal changes in HAI density for both wards are illustrated in Fig. 1.

## Discussion

### Principal findings

This study observed significantly divergent healthcare-associated infection (HAI) trajectories following HyTAMO coating application in a chronic respiratory care setting. The experimental ward demonstrated a progressive decline in HAI density from 4.70 to 0.90 per 1,000 patient-days over 5 months, whereas the control ward showed an initial decrease followed by a rebound to pre-intervention levels. The Poisson Group × Time interaction was statistically significant (one-tailed *p* = 0.038), supporting the interpretation that the intervention was associated with a differential trend in HAI incidence. However, given the non-randomized ward-level design, these findings should be interpreted as an association rather than definitive evidence of causality. Notably, this clinical signal emerged despite the absence of statistically significant differences in environmental bacterial counts during the monitored period (*p* = 0.076), highlighting a discrepancy between environmental and clinical outcomes that warrants further mechanistic investigation.

The divergent HAI trends observed in this study are consistent with prior reports of antimicrobial surface interventions, where reductions of approximately 21–58% have been described in acute care settings [12, 13]. In addition, the duration of the observed effect (~ 5 months) is comparable to, or longer than, those reported in prior real-world studies of antimicrobial surface interventions.

While direct comparisons should be interpreted cautiously due to differences in study design, setting, and patient populations, these findings suggest that antimicrobial coatings such as HyTAMO may have potential clinical relevance in chronic care environments, particularly where cleaning intensity and patient turnover differ from acute care settings.

### Interpretation of the environmental-clinical disconnect

A key finding of this study is the apparent discrepancy between environmental microbiological measurements and clinical infection outcomes. While no statistically significant Group × Time interaction was observed in environmental bacterial counts, a marked and sustained reduction in HAI incidence was observed in the experimental ward. This discrepancy represents a central observation of the study and warrants careful interpretation.

This inconsistency should not be interpreted as contradictory findings, but rather reflects the inherent limitations of conventional culture-based environmental measurements in capturing clinically relevant antimicrobial effects. Such methods primarily quantify total bacterial burden and do not distinguish between pathogenic and non-pathogenic organisms, nor do they capture microbial viability, virulence potential, or functional changes within microbial communities. Recent mechanistic studies suggest that antimicrobial surfaces may alter microbial redox balance, induce oxidative stress, and disrupt cellular integrity, thereby reducing microbial pathogenicity without necessarily affecting culturability [14]. In addition, emerging clinical evidence indicates that antimicrobial interventions may influence host–microbiome interactions and subclinical microbial activity in ways not detectable by conventional culture-based assays [15]. Together, these findings support the notion that culture-based enumeration alone may underestimate biologically relevant antimicrobial effects.

Several mechanisms may explain this observation. First, antimicrobial coatings may exert selective pressure on clinically relevant pathogens without substantially altering total bacterial counts. Although our 16 S rRNA sequencing suggested a reduction in the relative abundance of *Acinetobacter baumannii* and *Haemophilus influenzae*, these findings were based on relative abundance data and were not powered for formal quantitative comparisons. Exploratory classification of the sequencing results into pathogenic and commensal categories revealed a trend toward decreased pathogenic taxa and a proportional increase in commensal organisms in the experimental ward, though these differences did not reach statistical significance. Confirmation through targeted qPCR for key pathogens would strengthen these observations. Previous studies of antimicrobial surfaces have also suggested that selective ecological pressure may favor non-pathogenic species over clinically relevant pathogens [16]. Second, antimicrobial surfaces may affect microbial viability or virulence without necessarily reducing culturability. Viable-but-non-culturable (VBNC) states and sublethal cellular damage may reduce infection potential despite stable colony counts. Such effects cannot be captured by conventional culture-based methods and would require molecular approaches, such as viability PCR (e.g., PMA-qPCR), for further evaluation [17, 18]. Third, biofilm-related mechanisms may contribute to the observed clinical benefit. Antimicrobial coatings may inhibit biofilm formation or destabilize established biofilms, thereby reducing pathogen persistence and transmission risk even when planktonic bacterial counts remain unchanged. Fourth, the temporal mismatch between environmental monitoring and clinical surveillance should be considered when interpreting these findings. Environmental sampling was conducted for 8 weeks, whereas HAI surveillance continued for approximately 5 months. This difference was driven by operational constraints in the clinical setting, including ward-specific timing of terminal cleaning and the routine 3-month disinfection cycle. As environmental microbiological assessments were designed to capture short-term changes following coating application, they were limited to the period prior to the next scheduled terminal cleaning. In contrast, HAI surveillance is routinely performed on a monthly basis and allowed for extended follow-up beyond the environmental sampling window [19–22].

Consequently, direct temporal correlation between environmental microbial data and clinical outcomes across the entire observation period was not feasible. It is plausible that early changes in microbial characteristics—such as reductions in pathogen virulence, alterations in microbial community structure, or inhibition of biofilm formation—contributed to the sustained reduction in HAI incidence observed over time.

In addition to bacterial pathogens, the potential impact of antimicrobial coatings on opportunistic fungal infections should be considered. Patients requiring prolonged mechanical ventilation are at increased risk of secondary fungal infections, including invasive mold infections such as mucormycosis, particularly following viral respiratory illness. Environmental fungal spores represent an important source of these infections. Recent studies have suggested that environmental control strategies targeting microbial burden may also influence fungal exposure dynamics [23]. Although fungal outcomes were not assessed in this study, the potential for antimicrobial coatings to reduce fungal contamination and associated infection risk warrants further investigation.

Finally, the current microbiological analysis has inherent limitations that should be considered when interpreting the results. While total viable counts provide information on overall microbial burden, they do not differentiate between pathogenic and commensal organisms, and thus cannot reveal whether the coating exerts selective antimicrobial effects. The 16 S rRNA amplicon sequencing employed in this study provides semi-quantitative information on microbial community composition through relative abundance analysis; however, it does not yield absolute quantification of individual taxa. Our exploratory classification of sequencing results into pathogenic and commensal categories suggested a possible shift toward non-pathogenic taxa in the experimental ward, but these findings should be interpreted cautiously given the compositional nature of the data. Importantly, 16 S rRNA sequencing is inherently limited in its capacity to serve as a quantitative tool, as amplification biases and the compositional constraint of relative abundance data may obscure true changes in absolute microbial load. Quantitative real-time PCR (qPCR) targeting specific pathogenic species would provide more robust quantitative evidence and is recommended for future studies to validate the selective inhibitory effects suggested by the sequencing data. Additionally, future research should incorporate classification-based statistical analyses comparing pathogenic and commensal organism counts between groups, as well as viability assays (e.g., PMA-qPCR) and biofilm-specific analyses, to provide a more comprehensive mechanistic understanding.

Taken together, these findings suggest that environmental bacterial counts alone may not fully reflect the infection prevention effects of antimicrobial surface interventions, and underscore the importance of integrating microbiological and clinical outcome measures in future studies.

### Comparison with existing literature

Our findings both align with and differ from previous studies on antimicrobial surface interventions. Prior studies of copper surfaces have demonstrated concurrent reductions in environmental bacterial burden (approximately 0.7–2 log reduction) and HAI incidence (21–58% reduction), suggesting concordance between environmental microbiological and clinical outcomes in acute care settings [4, 12, 13]. For example, Salgado et al. reported a 0.76 log reduction in surface bacterial counts accompanied by a 58% decrease in HAI incidence in ICU environments [4]. In the present study, the experimental ward demonstrated a significant divergent trend in HAI incidence over a 5-month period (Group × Time interaction, one-tailed *p* = 0.038), with a progressive decline to 0.90 per 1,000 patient-days by the final month, despite the absence of a statistically significant Group × Time interaction in environmental bacterial counts during the monitoring period. This divergence suggests that clinical benefits may occur even in the absence of measurable reductions in total culturable bacterial burden. As discussed above, this finding is consistent with emerging mechanistic evidence indicating that antimicrobial surfaces may alter microbial viability, pathogenicity, or community composition without substantially reducing total colony counts.

With respect to durability, previous studies of silver-based antimicrobial surfaces have reported sustained antimicrobial activity for several months. Widmer et al. demonstrated approximately 6-month persistence of antimicrobial effects using silver-impregnated surface materials [24]. The ~ 5-month duration of reduced HAI incidence observed in this study is broadly consistent with these findings. However, many prior studies lacked concurrent control groups or systematic HAI surveillance, limiting causal interpretation. Although the present study is also subject to methodological limitations, the use of a parallel control ward combined with prospective HAI monitoring provides a more structured framework for evaluating clinical associations.

Recent systematic reviews have highlighted substantial methodological heterogeneity in antimicrobial surface research, including variability in study design, outcome measures, and analytical approaches [25, 26]. These reviews emphasize the need for standardized methodologies and integrated evaluation of both environmental and clinical endpoints. Our findings contribute to this body of literature by suggesting that environmental microbiological metrics and clinical infection outcomes may not always correlate directly, and should therefore be interpreted as complementary rather than interchangeable indicators of intervention effectiveness.

### Clinical and public health implications

The observed reduction in HAI incidence suggests that antimicrobial coatings such as HyTAMO may serve as a potential adjunctive strategy for infection prevention in chronic respiratory care settings.

Patients requiring prolonged mechanical ventilation represent a particularly vulnerable population, characterized by impaired host defenses, frequent use of invasive devices, and extended exposure to healthcare environments [27]. In such settings, where frequent and intensive environmental disinfection may be difficult to sustain, long-acting antimicrobial surface interventions may provide an additional layer of protection alongside standard infection control measures. In addition to bacterial infections, these patients are also at increased risk of opportunistic fungal infections, particularly following respiratory viral illness. Environmental fungal spores represent an important source of such infections, including invasive mold infections such as mucormycosis. Emerging evidence suggests that environmental control strategies targeting microbial burden may also influence fungal exposure dynamics. Although fungal outcomes were not assessed in this study, the potential for antimicrobial coatings to reduce environmental fungal contamination and associated infection risk warrants further investigation [23].

From a health system perspective, reductions in HAI incidence in long-term care populations may translate into meaningful decreases in healthcare burden, given the prolonged length of stay and high treatment costs associated with infections in this population [28]. Even modest reductions in infection rates may result in cumulative benefits at the system level. Future studies should incorporate formal cost-effectiveness analyses to evaluate the balance between coating implementation costs and potential reductions in infection-related healthcare utilization.

### Strengths of the study

This study has several notable strengths. First, the extended duration of patient stay in the study population (mean approximately 1164 days, or 3.2 years) provided a unique opportunity to evaluate the persistence of antimicrobial coating effects under real-world conditions. Compared with acute care settings characterized by high patient turnover, this relatively stable population reduces variability related to patient replacement and allows for a more consistent assessment of long-term intervention effects.

In addition, the chronic respiratory care setting may reduce confounding related to cleaning intensity. Previous studies have suggested that frequent and intensive disinfection in acute care environments may obscure the measurable effects of antimicrobial surface interventions [20, 21]. In contrast, the standardized once-daily cleaning protocol applied in both wards in this study may have allowed the effects of the coating to be more readily observed while maintaining routine infection control practices.

Second, the use of a parallel control ward combined with prospective HAI surveillance provides a structured framework for evaluating clinical outcomes in relation to environmental interventions. Although the non-randomized design introduces potential limitations, the consistent application of clinical care protocols and cleaning procedures across both wards supports the internal comparability of the study.

Finally, the age difference between wards (80.0 vs. 75.6 years) represents a baseline characteristic that would typically be associated with increased infection risk in the experimental group [20]. The observation of reduced HAI incidence despite this imbalance suggests that the observed association is unlikely to be explained solely by patient-level risk differences, although residual confounding cannot be excluded.

### Limitations and future research

Several important limitations should be acknowledged. First, the non-randomized allocation at the ward level (2 F vs. 3 F) represents an inherent methodological limitation and introduces the possibility of residual confounding. Although efforts were made to standardize environmental conditions, clinical practices, and cleaning protocols across both wards, unmeasured differences—such as airflow dynamics or microenvironmental variation—cannot be fully excluded. In addition, complete blinding was not feasible due to the nature of the environmental intervention. However, HAI surveillance was conducted using standardized CDC criteria by trained infection control personnel, which helps reduce outcome assessment bias. Second, the temporal mismatch between environmental monitoring and clinical surveillance should be considered. Environmental sampling was limited to 8 weeks, whereas HAI surveillance extended to approximately 5 months. This difference reflects real-world operational constraints, including ward-specific timing of terminal cleaning and the routine 3-month disinfection cycle. As a result, direct temporal correlation between environmental microbiological data and clinical outcomes across the full observation period was not possible. Third, the use of pooled environmental sampling may have obscured site-specific variability in microbial contamination. In addition, the single-center design limits the generalizability of the findings to other healthcare settings with different patient populations, environmental conditions, or infection control practices. Fourth, the microbiological methods used in this study—total viable counts and 16 S rRNA sequencing—have inherent limitations. While these approaches provide information on overall microbial burden and community composition, they do not fully capture microbial viability, functional activity, or pathogen-specific dynamics. In particular, the lack of quantitative molecular analyses limits the ability to assess selective effects on pathogenic organisms. Future studies incorporating quantitative molecular techniques (e.g., qPCR), viability assays (e.g., PMA-qPCR), and biofilm-specific analyses would provide more comprehensive insight into underlying mechanisms. Finally, this study did not assess fungal contamination or fungal infection outcomes. Given the potential role of environmental fungi in opportunistic infections among ventilator-dependent patients, future research should include fungal surveillance to more comprehensively evaluate the clinical impact of antimicrobial coatings.

Overall, these findings should be considered hypothesis-generating and require confirmation in randomized controlled studies with integrated microbiological and clinical outcome assessments.

## Conclusions

In this prospective controlled study conducted in chronic respiratory care wards, application of the HyTAMO titanium dioxide–silver composite coating was associated with significantly divergent HAI trajectories compared with the control ward over an approximately 5-month period. The experimental ward showed a progressive decline in HAI density from 4.70 to 0.90 per 1,000 patient-days, while the control ward exhibited a rebound to pre-intervention levels (Poisson Group × Time interaction, one-tailed *p* = 0.038). Notably, this clinical signal was observed despite the absence of statistically significant differences in environmental bacterial counts during the initial monitoring period (*p* = 0.076). This finding highlights a potential disconnect between conventional culture-based environmental measurements and clinically relevant antimicrobial effects, suggesting that mechanisms beyond simple reductions in total bacterial burden—such as alterations in microbial viability, pathogenicity, or biofilm dynamics—may contribute to infection risk reduction.

The chronic care setting, characterized by prolonged patient stays (mean approximately 3.2 years) and relatively stable environmental conditions, provided a unique context for evaluating the persistence of antimicrobial coating effects under real-world conditions, with reduced confounding from patient turnover commonly encountered in acute care environments. These findings suggest that antimicrobial coatings may have potential value as an adjunctive infection prevention strategy in long-term care settings, particularly where intensive and frequent environmental cleaning may be difficult to sustain.

However, given the non-randomized study design, potential residual confounding, and limitations in microbiological methods and temporal alignment between environmental and clinical data, these results should be interpreted with caution and considered hypothesis-generating rather than definitive evidence of causality.

Future studies incorporating randomized controlled designs, extended environmental monitoring aligned with clinical surveillance, and advanced microbiological analyses—including quantitative molecular techniques, viability assays, and biofilm assessment—are warranted to confirm causality and further elucidate underlying mechanisms. In addition, evaluation of fungal contamination and infection outcomes may provide a more comprehensive understanding of the clinical impact of antimicrobial surface interventions in high-risk populations.

## Data Availability

HAI rate and patient composition data supporting the conclusions of this article are presented in full within the manuscript. Environmental sampling data (CFU and ATP values) are held by the Industrial Technology Research Institute (ITRI) and are available upon reasonable request to Dr. Wen-Ching Sun. Raw 16 S rRNA sequencing reads have been deposited in the NCBI BioProject database under accession number PRJNA1462855 (https://www.ncbi.nlm.nih.gov/bioproject/1462855).

## Abbreviations

ATP: Adenosine triphosphate
CDC: Centers for Disease Control and Prevention
CFU: Colony-forming units
CI: Confidence interval
HAI: Healthcare-associated infection
HyTAMO: Hybrid TiO₂-Ag Micro-Organism Shield
ICU: Intensive care unit
IRR: Incidence rate ratio
RLU: Relative light units
VBNC: Viable-but-non-culturable
qPCR: Quantitative polymerase chain reaction
PMA-qPCR: Propidium monoazide quantitative polymerase chain reaction
rRNA: Ribosomal ribonucleic acid
TiO₂: Titanium dioxide
Ag: Silver
